# The effect of seasonal changes and climatic factors on suicide attempts of young people

**DOI:** 10.1186/s12888-017-1532-7

**Published:** 2017-11-15

**Authors:** Türkan Akkaya-Kalayci, Benjamin Vyssoki, Dietmar Winkler, Matthaeus Willeit, Nestor D. Kapusta, Georg Dorffner, Zeliha Özlü-Erkilic

**Affiliations:** 1 Outpatient Clinic of Transcultural Psychiatry and Migration Induced Disorders in Childhood and Adolescence, Department of Child and Adolescent Psychiatry, Medical University of Vienna, Währinger Gürtel 18-20, 1090 Vienna, Austria; 2 Department for Psychiatry and Psychotherapy, Clinical Division of Social Psychiatry, Medical University of Vienna, Währinger Gürtel 18-20, 1090 Vienna, Austria; 3 Department of Psychoanalysis and Psychotherapy, Medical University of Vienna, Währinger Gürtel 18-20, 1090 Vienna, Austria; 4 Section of Artificial Intelligence, Center for Medical Statistics, Informatics and Intelligent Systems, Medical University of Vienna, Freyung 6/2, 1010 Vienna, Austria

**Keywords:** Suicide attempts, Adolescents, Seasonal changes, Climatic variations, Temperature

## Abstract

**Background:**

Seasonal changes and climatic factors like ambient temperature, sunlight duration and rainfall can influence suicidal behavior.

**Methods:**

This study analyses the relationship between seasonal changes and climatic variations and suicide attempts in 2131 young patients in Istanbul, Turkey.

**Results:**

In our study sample, there was an association between suicide attempts in youths and seasonal changes, as suicide attempts occurred most frequently during summer in females as well as in males. Furthermore, there was a positive correlation between the mean temperature over the past 10 days and temperature at the index day and suicide attempts in females. After seasonality effects were mathematically removed, the mean temperature 10 days before a suicide attempt remained significant in males only, indicating a possible short-term influence of temperature on suicide attempts.

**Conclusions:**

This study shows an association between suicide attempts of young people and climatic changes, in particular temperature changes as well as seasonal changes. Therefore, the influence of seasonal changes and climatic factors on young suicide attempters should get more attention in research to understand the biopsychosocial mechanisms playing a role in suicide attempts of young people. As suicide attempts most frequently occur in young people, further research is of considerable clinical importance.

## Background

Seasonal effects are known to influence the frequency of committed suicides throughout the year as well as the number of suicide attempts [1, 2]. A suicide peak in spring was observed in countries of the northern and southern hemisphere [1, 3, 4]. Studies could further show an effect of climatic factors on suicide attempts [3, 5–7], as well as on completed suicides [3, 8, 9]. Temperature, humidity [4], the length of the day and intensity of sunlight [1, 3, 4] were found to influence suicidal behavior in large population-based studies. It was proposed that sunshine, through interaction with the serotonergic systems, is of particular importance in this regard. Even short-term changes in lighting conditions can affect depression-like behaviour in rodents and lead to changes in brain monoamine transmission [10]. Also a higher ambient temperature has already been assumed to be involved in serotoninergic neurotransmitter systems leading to impulsiveness and aggression, presumably influencing suicide [11–13]. Previous studies report a positive association between suicide and higher ambient temperature, which is mainly increased in males and those of older age [11, 14].

The influence of ambient temperature and sunshine on suicidal behavior to our knowledge has not yet been investigated in children and adolescents. As suicide attempts most frequently occur in adolescents and young adults aged between 15 and 25 [5, 15, 16], further research is of paramount importance from a clinical point of view [17].

Therefore, the present study is the first one examining the influence of seasonal changes and climatic variations on young suicide attempters. Our aim was to analyse the influence of seasonal changes as well as temperature and two other climatic indicators (daylight duration and rainfall) on attempted suicides in young people up to age 25.

## Methods

This study is a retrospective analysis of suicide attempt reports for patients between ages 15 and 25 (*N* = 2131) during a one-year period (from January 1st, 2010, to December 31st, 2010). The present study was conducted in Istanbul, one of the big metropolitan regions of the world, with approximately 17 million inhabitants and an area of 5343.02 km^2^ [18].

The case records were collected from all state hospitals in Istanbul by the Health Directorate of Istanbul, a department of the Turkish Ministry of Health. For each of the 365 days of the year 2010 the number of suicide attempts for males and females were available. Data on the climatic indicators temperature (°C), sunshine duration (hours), total rainfall amount (mm) for Istanbul for the year 2010 were obtained from the General Meteorological Department of Ankara (Turkey), also on a daily basis.

There were temperature variations across the different areas of Istanbul. As we analyse whole temperature variations of overall Istanbul, we have to take the average temperature.

We first analysed this data for seasonal effects by summarizing suicide attempts by season, for males and females separately. A chi-squared test was used to test for differences between men and women.

We then analysed the relationship between the suicide attempts and the different climatic indicators by correlating the number of suicide attempts with climatic variables on the same day (index day), and with their means over 10 days before the suicide attempt. To account for possible nonlinearities or outliers, we used Spearman’s correlation coefficients in all calculations.

Since all variables considered – temperature, sunlight duration and amount of rainfall, but also the number of suicide attempts – can be expected to show a seasonal effect, in a second analysis seasonality was removed using a trigonometric sine model (exactly one period) for the dependency between the day of the year and the variable. After removal of seasonality all correlations were calculated again to examine short-term influences between climatic factors and suicide attempts beyond known seasonal effects.

We used “SPSS 21” [19] for statistical analysis and Matlab [Mathworks] for trigonometric model fitting. A basic significance level of 0.05 was corrected by the Bonferroni method to account for multiple testing within each set of research questions.

## Results

### Seasons

In the data set used, the majority of the 2131 suicide attempters were females (81.7%), *N* = 1740; whereas only *N* = 391 (18.3%) of suicide attempters were males.

**Fig. 1:**
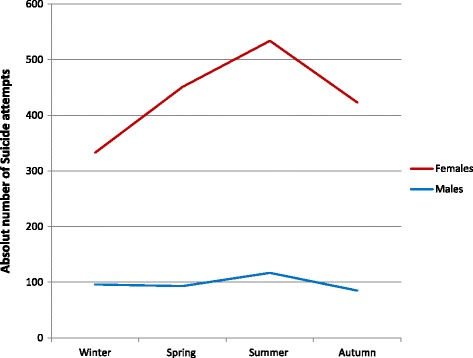
Distribution of suicide attempts by sex and season

Figure 1 shows the distribution for male and female attempts over the year by meteorological season. The most frequent suicide attempts occurred in summer (June–August, 30.6%) followed by spring (March–May, 25.4%), autumn (September–November, 23.8%) and winter (December–February, 20.1%). Testing this distribution against the null hypothesis, that the percentage of suicide attempts is the same for all seasons, points to a highly significant seasonality effect (Chi-squared test, χ^2^ = 53.803, df = 3, *p* < 0.0001).

### Season and gender

There was no significant sex difference (Chi-squared test, χ^2^ = 6.1, df = 3, *p* = 0,107) concerning the seasonal changes.

### Correlation with climatic factors

Table 1 shows the Spearman correlation coefficients expressing the correlations between amount of rainfall, temperature and sunlight duration on a particular day (index day), or as a mean over the 10 days preceding the index day, on the one hand, and male and female suicide attempts, on the other. Since in some days, one or more of the climatic variables are missing, the actual n for each case is also listed. Since 24 tests are applied altogether, the significance level was adjusted to α = 0.05/24 = 0.0021.

**Table 1:** Spearman correlation between climatic factors, either on the index day or as a mean of the 10 previous days, and suicide attempts, by sex

With seasonal pattern		Female suicide attempts		Male suicide attempts	
	n	r	*p*	r	*p*
Amount of rainfall, index day	365	0.016	0.760	0.027	0.603
Amount of rainfall, mean of 10 previous days	355	0.085	0.110	−0.053	0.857
Temperature, index day	364	0.213**	0.000	0.009	0.857
Temperature, mean of 10 previous days	345	0.189**	0.000	0.050	0.359
Sunlight duration, index day	307	0.097	0.090	−0.126*	0.028
Sunlight duration, mean of 10 previous days	264	0.077	0.210	−0.025	0.690

*denotes significance at the original level of α = 0.05

**denotes statistical significance after Bonferroni correction (*p* < 0.0021)

The main significant correlations after Bonferroni correction are a positive correlation between the temperature on the index day and female suicide attempts (*r* = 0.231), as well as between the mean temperature over the past 10 days and female suicide attempts (*r* = 0.189).

We further performed a post-hoc partial correlation, controlling the correlation between temperature and female suicide attempts using the factor of radiation, as measured by sunlight duration. As a result, the correlation between the temperature on the index day and the suicide attempts reduced from *r* = 0.213 to *r* = 0.159 (*p* = 0.005), while the correlation between the temperature on the last 10 days and the suicide attempts vanished (*r* = 0.073, *p* = 0.236).

Even when controlling for covariates (i.e. radiation as measured by sunlight duration) we were able to ascertain a significant correlation between temperature and female suicide attempts on the index day.

We also calculated a regression model for the highly significant correlation between temperature on the index day and female suicide attempts, leading to a coefficient of 0.08 (*p* < 0.001). In other words, the number of female suicide attempts tends to increase by 0.8 on average per 10 degrees Celsius increase in temperature.

### Correlation with climatic factors after removal of seasonality

**Fig. 2:**
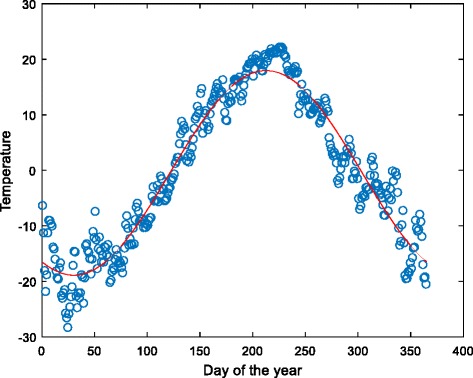
The seasonality of temperature depicted as a dependency on the day of the year, which was modelled using trigonometric (sine) function. The residuals around that function then represent the new values after removal of seasonality

The seasonality of the number of suicide attempts was confirmed by the previous results. All four climatic variables also showed a dependence on the day of the year, with a peak or trough in summer, which was modelled by a trigonometric regression function (sine), as depicted for temperature in Fig. 2.

Table 2 presents the correlation results, in analogy to Table 1, after removal of seasonality from all variables including the number of suicide attempts.

**Table 2:** Correlation between climatic factors after removal of seasonality, either on the index day or as a mean of the 10 previous days, and suicide attempts, by sex, also after removal of seasonality

After removal of seasonality		Female suicide attempts		Male suicide attempts	
	n	r	*p*	r	*p*
Amount of rainfall, index day	364	−0.020	0699	0.124*	0.017
Amount of rainfall, mean of 10 previous days	355	0.061	0.255	0.043	0.417
Temperature, index day	364	0.035	0.509	−0.006	0.908
Temperature, mean of 10 previous days	345	−0.028	0.611	0.098	0.069
Sunlight duration, index day	307	−0.035	0.537	−0.132*	0.021
Sunlight duration, mean of 10 previous days	264	−0.131*	0.033	−0.014	0.824

* denotes significance at the original level of α = 0.05

** denotes statistical significance after Bonferroni correction (*p* < 0.0021)

After Bonferroni correction, none of the correlations depicted remain significant.

## Discussion

The aim of this study was to assess the relationship between meteorological factors and the incidence of suicide attempts in a large Turkish sample of suicide attempters. There are two main findings in this study. First, our analysis shows a positive correlation between temperature and suicide attempts in females, before the removal of seasonality effects. Therefore, temperature seems to be the main seasonal effect behind female suicide attempts in our sample. This is underlined by the observation that despite a relatively high intercorrelation between the climatic variables, the other meteorological variables included do not show the same significant associations with suicide attempts. Because seasonality might be spuriously correlated with other important behavioral or social variables not accounted for in this study, it remains open whether temperature has any causal effects on female suicide attempts. This hypothesis was not supported by daily level data.

The second main finding is a positive correlation between temperature and suicide attempts in males, after the mathematical removal of seasonality. This result, more convincingly than any possible seasonal correlation, is a stronger support for a direct effect of temperature in triggering male suicide attempts. Correlations of other meteorological variables with suicide attempts in males even go in the opposite direction (such as a slightly negative correlation between de-seasonalized sunlight duration and male suicide attempts). However, none of these correlations remained significant after Bonferroni correction.

The difference between correlations of values before removal of seasonality and correlations after such removal can be interpreted as follows. Any correlation in the former case could be the result of a major hidden seasonal effect and thus does not necessarily point to any direct relationship between the variables. In the latter case, however, we mainly look at the differences of each variable from an average value at a certain time during the year, and their correlations. So, for instance, the significant positive correlation (*r* = 0.174) between the mean temperature of 10 previous days and male suicide attempts, can be understood as follows: a deviation of temperature over a period of 10 days from the seasonal average is positively correlated to the deviation of the number of male suicide attempts from the average at the same point in time. In other words, 10-day periods of warmer temperatures than usual – whether they are measured against relatively low averages in winter or against relatively high averages in summer – tend to correlate with higher than average suicide attempt rates. Since the main seasonal factor is removed from this analysis, a potential causality between the variables is more likely than in the correlations of the original time series.

The positive relationship between temperature and the number of completed suicides has been previously reported in a number of studies [1, 11, 20–30]. Our results extend this claim to suicide attempts, although we share the view that there is still no agreement about the possible biological mechanism behind the association between temperature and suicidal behaviour and some researchers call for the need to examine both sunlight and temperature in studies on suicide on a daily base [31]. One common hypothesis is that an increase in suicidal behaviour in summer may be explained by seasonal changes in the serotonin system, which increases impulsiveness and aggression and consequently the risk for suicidal behaviour [12, 32], which might also hold true for suicide attempts. However, other explanations hypothesize that an increased activity of brown adipose tissue (BAT) may also play a crucial role in suicidal behaviour [23], as hyperactivity of BAT was reported in depressed persons who completed suicide [33]. The primary function of BAT is thermoregulation and the regulation of metabolism [34]. BAT enhances cold tolerance by reducing heat tolerance and increasing anxiety and psychomotor agitation. Consequently, BAT influences mood negatively. As BAT is influenced by temperature changes, it is overactive in suicide completers with prior depressive episodes [23]. Temperature changes also enhance the metabolic activity of BAT and may consequently increase the risk of suicidal behaviour over a persistent time [35]. Furthermore, BAT is present over all ages of human beings and excessively in adolescents [36]. Comparable to sunlight, also ambient temperature has been reported to be involved in the serotonin system influencing suicide rates [11, 27]. We suppose that ambient temperature changes, besides BAT modulation presumably also influence the serotonin neurotransmission in young people leading to impulsivity and suicide attempts [37, 38]. Our results are in line with the abovementioned hypothesis, and show that there is a positive association between higher ambient temperature and the number of suicide attempts in young males on a daily level, independent of seasonal effects.

## Conclusions

Although there were no gender differences in the seasonality of suicide attempts, there was an association between suicide attempts and seasons in the present study sample, as suicide attempts of young people most frequently occurred in summer. In concordance to these results, previous studies have shown an association between seasons and suicide attempts [39, 40] as well as completed suicides [41]. Further, in line with previous studies, we found that the numbers of female suicide attempters peaked in summer [6] and spring [8] with the lowest incidence in winter [40].

Similar to prior studies [2, 5, 6, 8, 15, 16, 42–45], this study confirms that suicide attempts are committed more frequently by females than males. The WHO/EURO multicentre study on para-suicide which analysed suicide attempts in 15 European countries showed the number of female suicide attempters to be higher in all but one study centre [46]. In contrast to that, completed suicides are much more common in males [2, 43].

Although suicide attempts most frequently occur in adolescents and young adults between ages 15 and 25 [5, 15, 16], available data concerning suicidal behavior, especially suicide attempts of young people are quite limited. This influences our understanding of bio-psychosocial mechanisms of suicidal behavior and currently limits effective preventive health-care measures in this field [21, 47–49]. Since young people need a more rigorous follow-up care after a suicide attempt and data show that they do not receive this required professional support [50], developing the necessary preventive health-care measures is an imperative [47, 50–58].

Hancock, et al. (2011) report a strong association of various sets of genes with climate variables. One set is central in the differentiation of adipocytes in brown adipose tissue. BAT is essential for natural selection and therefore a disrupted BAT activity may have an impact on adaptation and subsequently on survival [59]. Some researchers even suggest that the constant increase in climatic warming will lead to hyperactivity of BAT and as a consequence will augment the mortality due to suicide within vulnerable populations [23]. Whether this hypothesis will hold true is to be elucidated in future studies. However, increasing evidence and our results show that the climatic factors and seasonal changes play a role in in young suicide attempters and thus should get more attention in research and in suicide prevention programs.

### Limitations

In the present study, only suicide attempts by youth up to age 25 were considered, therefore, the results are not representative for the whole population in Istanbul.

Furthermore, data from only one year was available, leading to low numbers, thus the robustness of the results should be examined in a longer time series.

The ratio of suicide attempts to actual suicides varies widely and is difficult to study because there is no systematic national registry on suicide attempts across Turkey.

Intra-individual and psychosocial factors that can influence suicidal behavior were not considered in our study thus limiting the possibilities of further interpretations [60–63].

## Abbreviation

BAT: Brown adipose tissue
